# Fiber-coupled light-emitting diodes (LEDs) as safe and convenient light sources for the characterization of optoelectronic devices

**DOI:** 10.12688/openreseurope.14018.1

**Published:** 2021-08-23

**Authors:** Jorge Quereda, Quinghua Zhao, Enrique Diez, Riccardo Frisenda, Andrés Castellanos-Gomez

**Affiliations:** 1 Nanotechnology Group, USAL–Nanolab, Univesidad de Salamanca, Salamanca, Junta de Castilla y León, 37007, Spain; 2 Materials Science Factory, Instituto de Ciencia de Materiales de Madrid (ICMM-CSIC), Madrid, Madrid, 28049, Spain

**Keywords:** Optoelectronics, Photocurrent spectroscopy, 2D materials

## Abstract

Optoelectronic device characterization requires to probe the electrical transport changes upon illumination with light of different incident powers, wavelengths, and modulation frequencies. This task is typically performed using laser-based or lamp + monochromator-based light sources, that result complex to use and costly to implement. Here, we describe the use of multimode fiber-coupled light-emitting diodes (LEDs) as a simple, low-cost alternative to more conventional light sources, and demonstrate their capabilities by extracting the main figures of merit of optoelectronic devices based on monolayer MoS _2_, i.e. optical absorption edge, photoresponsivity, response time and detectivity. The described light sources represent an excellent alternative for performing optoelectronic characterization experiments on a limited budget.

## Plain language summary

In this work we present a low-cost light source based on light-emitting diodes (LEDs) for its use in measurement systems for characterization of photodetectors. The reduced cost and ease of use of the proposed light source makes it ideal for device characterization experiments on a limited budget. 

## Introduction

The scientific activity on optoelectronics has grown steadily over the last decades thanks to the discovery of novel promising materials such as two-dimensional transition metal dichalcogenides ^1– 7^, perovskites ^8– 14^, etc. Indeed, according to the Dimensions database, the number of scientific publications in this field has grown from roughly 4000 articles per year in 1990 to more than 60000 in 2020.

To characterize the main figures of merit of optoelectronic devices accurately and quantitatively, it is necessary to probe the electrical transport changes upon illumination with light of different incident powers, wavelengths, and modulation frequencies, which typically require the use of specialized light sources. In most laboratories, free-space laser sources are commonly used to characterize the response to light of the fabricated devices. While these light sources present several advantages (e.g., they are very bright, and have very well-defined wavelengths) they also have some shortcomings (high cost, safety issues, speckle, and restringing mounting conditions amongst others). Moreover, to achieve the required functionality, laser-based or lamp + monochromator-based testing setups usually must be combined with additional optical elements, such as neutral density filters to change the intensity of the incident light or mechanical choppers to modulate the incident signal in time, adding to their prize and complexity of usage. Therefore, an alternative cost-efficient light source, fiber-coupled, without speckle and with a fully voltage-based adjustment of the illumination intensity and modulation frequency would be highly desirable.

In our laboratory we started seven years ago to employ multimode fiber-coupled light emitting diode (LED) sources to test optoelectronic devices and they have proven to be a very convenient alternative to the commonly used free-space laser or lamp based light sources. In fact, we have found that these light sources are very simple to operate and can be readily used to extract figures-of-merit of 2D based photodetectors, without presenting the safety issues associated to the use of free-space high power collimated laser beams. During this time, we have also seen that their use is not widely spread, at least in the community working on 2D materials based optoelectronic devices, motivating us to write this paper.

In this work, we thoroughly describe the use of fiber-coupled LED light sources to extract the different figures of merit of photodetector devices. We illustrate their use by characterizing a single-layer MoS _2_ photodetector and show how one can easily extract relevant optoelectronic parameters such as the response time, photocurrent power dependence, responsivity and detectivity. The capability of the system to accurately extract these parameters at different wavelengths allows us to accurately characterize the responsivity spectrum of the device, even when using other lights sources with a non-constant spectral power density.

The presented fiber-coupled LEDs are rather inexpensive, if compared with laser systems, and are modular making it possible to improve the system little by little. We hope that this Method article can be of interest of the researchers that are setting up their laboratories, especially those running under a tight budget.

## Methods

### Fiber-coupled LED light sources

**Figure 1.**
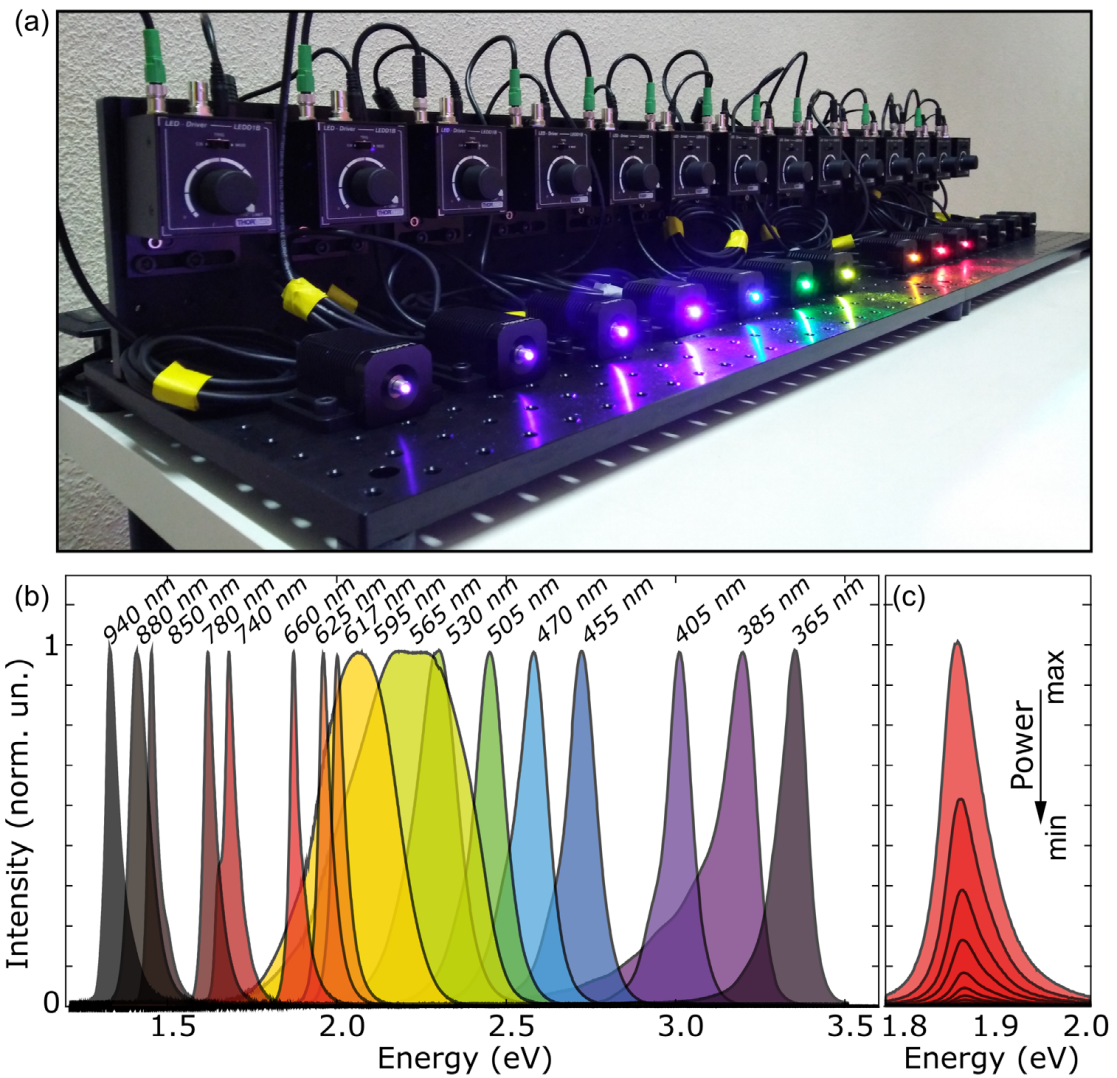
Bench with an assemble of fiber-coupled light-emitting diodes (LEDs). ( **a**) Picture of an illumination system implemented with 14 different fiber-coupled LEDs with their power supplies. ( **b**) Spectra of 17 different LED light sources used in our laboratory. ( **c**) Spectra of a 660 nm LED source at different biasing conditions to control the intensity of the out-coming light.

Our LED light sources are based on the Thorlabs MXXXF fiber-coupled LEDs, the Thorlabs LEDD1 controller and a programmable bench power supply (TENMA 72-2705). Figure 1a shows a picture of several LEDs sources with their controllers, mounted on dedicated breadboards that can be moved around the laboratory. Table 1 summarizes the different components used for the assembly of our fiber-coupled LEDs based light sources. Note that one can decide the number of different LED modules to add, with an average total cost of ~800€ per LED with different wavelengths. To illuminate our devices, a multimode optical fiber is attached to the LED source of the desired wavelength and the other end of the fiber is attached to a lens system placed above the optoelectronic device under study. By placing the core of the fiber at the image plane of the lens system we project an image of the core onto the device under study. Unlike with focused free-space laser sources, this method yields circular spots with homogeneous power density and (because of the use of incoherent sources) speckle-free ^15^. This is highly desirable to facilitate the calculation of the incident power and thus to accurately determine the figures of merit of photodetectors.

**Table 1.** List of required components for the assembly of the fiber-coupled light emitting diodes (LEDs) based light sources.

	Code	Description	Unitary price	Units	Total price
**UV to BLUE bench**	**MB3060/M**	Aluminum Breadboard, 300 mm x 600 mm x 12.7 mm, M6 Taps	248.11 €	1	248.11 €
	**MB1560/M**	Aluminum Breadboard, 150 mm x 600 mm x 12.7 mm, M6 Taps	143.25 €	1	143.25 €
	**AP90L/M**	Large Right-Angle Mounting Plate, M6 x 1.0 Compatible	149.46 €	1	149.46 €
	**RDF1**	Rubber Damping Feet, Set of 4	4.92 €	1	4.92 €
	**M365FP1**	365 nm, 9.8 mW (Min) Fiber-Coupled LED, 1400 mA, SMA	601.99 €	1	601.99 €
	**M385FP1**	385 nm, 18 mW (Min) Fiber-Coupled LED, 1400 mA, SMA	601.99 €	1	601.99 €
	**M405FP1**	405 nm, 19.3 mW (Min) Fiber-Coupled LED, 1400 mA, SMA	601.99	1	601.99
	**M420F2**	Violet (420 nm) Fiber-Coupled LED, SMA, 1000 mA, 8.90 mW (Min)	394,20 €	1	394,20 €
	**M455F1**	455 nm, 17 mW (Min) Fiber-Coupled LED, 1000 mA, SMA	388,55 €	1	388.55 €
	**KPS101**	15 V, 2.4 A Power Supply Unit with 3.5 mm Jack Connector for One K- or T-Cube	32.14 €	5	160.70 €
	**LEDD1B**	T-Cube LED Driver, 1200 mA Max Drive Current (Power Supply Not Included)	294.11 €	5	1 764.66 €
	Code	Description	Unitary price	Units	Total price
**BLUE to RED bench**	**MB3060/M**	Aluminum Breadboard, 300 mm x 600 mm x 12.7 mm, M6 Taps	248.11 €	1	248.11 €
	**MB1560/M**	Aluminum Breadboard, 150 mm x 600 mm x 12.7 mm, M6 Taps	143.25 €	1	143.25 €
	**AP90L/M**	Large Right-Angle Mounting Plate, M6 x 1.0 Compatible	149.46 €	1	149.46 €
	**RDF1**	Rubber Damping Feet, Set of 4	4.92 €	1	4.92 €
	**M470F1**	Blue (470 nm) Fiber-Coupled LED, SMA, 1000 mA, 8.0 mW (Min)	339,30 €	1	339.30 €
	**M505F1**	Cyan (505 nm) Fiber-Coupled LED, SMA, 1000 mA, 7.0 mW (Min)	339,30 €	1	339.30 €
	**M530F2**	530 nm, 6.8 mW (Min) Fiber-Coupled LED, 1000 mA, SMA	371,82 €	1	371.82 €
	**M565F3**	565 nm, 9.9 mW (Min) Fiber-Coupled LED, 700 mA, SMA	410.09 €	1	410.09 €
	**M595F2**	595 nm, 8.7 mW (Min) Fiber-Coupled LED, 1000 mA, SMA	360,99 €	1	360.99 €
	**M617F2**	617 nm, 10.2 mW (Min) Fiber-Coupled LED, 1000 mA, SMA	371,82 €	1	371.82 €
	**M625F2**	625 nm, 13.2 mW (Min) Fiber-Coupled LED, 1000 mA, SMA	360.99 €	1	360.99 €
	**KPS101**	15 V, 2.4 A Power Supply Unit with 3.5 mm Jack Connector for One K- or T-Cube	32.14 €	7	224.98 €
	**LEDD1B**	T-Cube LED Driver, 1200 mA Max Drive Current (Power Supply Not Included)	294.11 €	7	2 058.77 €
	Code	Description	Unitary price	Units	Total price
**RED to NIR bench**	**MB3060/M**	Aluminum Breadboard, 300 mm x 600 mm x 12.7 mm, M6 Taps	248.11 €	1	248.11 €
	**MB1560/M**	Aluminum Breadboard, 150 mm x 600 mm x 12.7 mm, M6 Taps	143.25 €	1	143.25 €
	**AP90L/M**	Large Right-Angle Mounting Plate, M6 x 1.0 Compatible	149.46 €	1	149.46 €
	**RDF1**	Rubber Damping Feet, Set of 4	4.92 €	1	4.92 €
	**M660F1**	Deep Red (660 nm) Fiber-Coupled LED, SMA, 1000 mA, 13.0 mW (Min)	339,30 €	1	339.30 €
	**M740F2**	740 nm, 4.1 mW (Min) Fiber-Coupled LED, 800 mA, SMA	426,88 €	1	426.88 €
	**M780F2**	780 nm, 5.5 mW (Min) Fiber-Coupled LED, 800 mA, SMA	378,70 €	1	378.70 €
	**M850F2**	IR (850 nm) Fiber-Coupled LED, SMA, 1000 mA, 10.5 mW (Min)	339,30 €	1	339.30 €
	**M880F2**	IR (880 nm) Fiber-Coupled LED, SMA, 1000 mA, 10.5 mW (Min)	378,70 €	1	378.70 €
	**M940F3**	940 nm, 10 mW (Min) Fiber-Coupled LED, 1000 mA, SMA	363.60 €	1	363.60 €
	**M1050F1**	1050 nm, 1.1 mW (Min) Fiber-Coupled LED, 700 mA, SMA	426,88 €	1	426.88 €
	**KPS101**	15 V, 2.4 A Power Supply Unit with 3.5 mm Jack Connector for One K- or T-Cube	32.14 €	7	224.98 €
	**LEDD1B**	T-Cube LED Driver, 1200 mA Max Drive Current (Power Supply Not Included)	294.11 €	7	2 058.77 €

Figure 1b shows the individual spectra of the 17 LED sources, spanning the spectral range from 365 nm to 940 nm. Each of the LEDs emits light at a different, narrow, spectral window, with full-width-at-half-maximum ranging from ~10 nm to ~100 nm (depending on the wavelength, the typical value is ~30 nm). The intensity of the emitted light can be tuned, either manually or via an external voltage input. Figure 1c shows the spectral emission profile of an individual LED at different operation powers. While the spectral profile slightly changes with the power, the emission peak remains stable within a 5 meV range.

## Results

### Device fabrication and optoelectronic response

**Figure 2.**
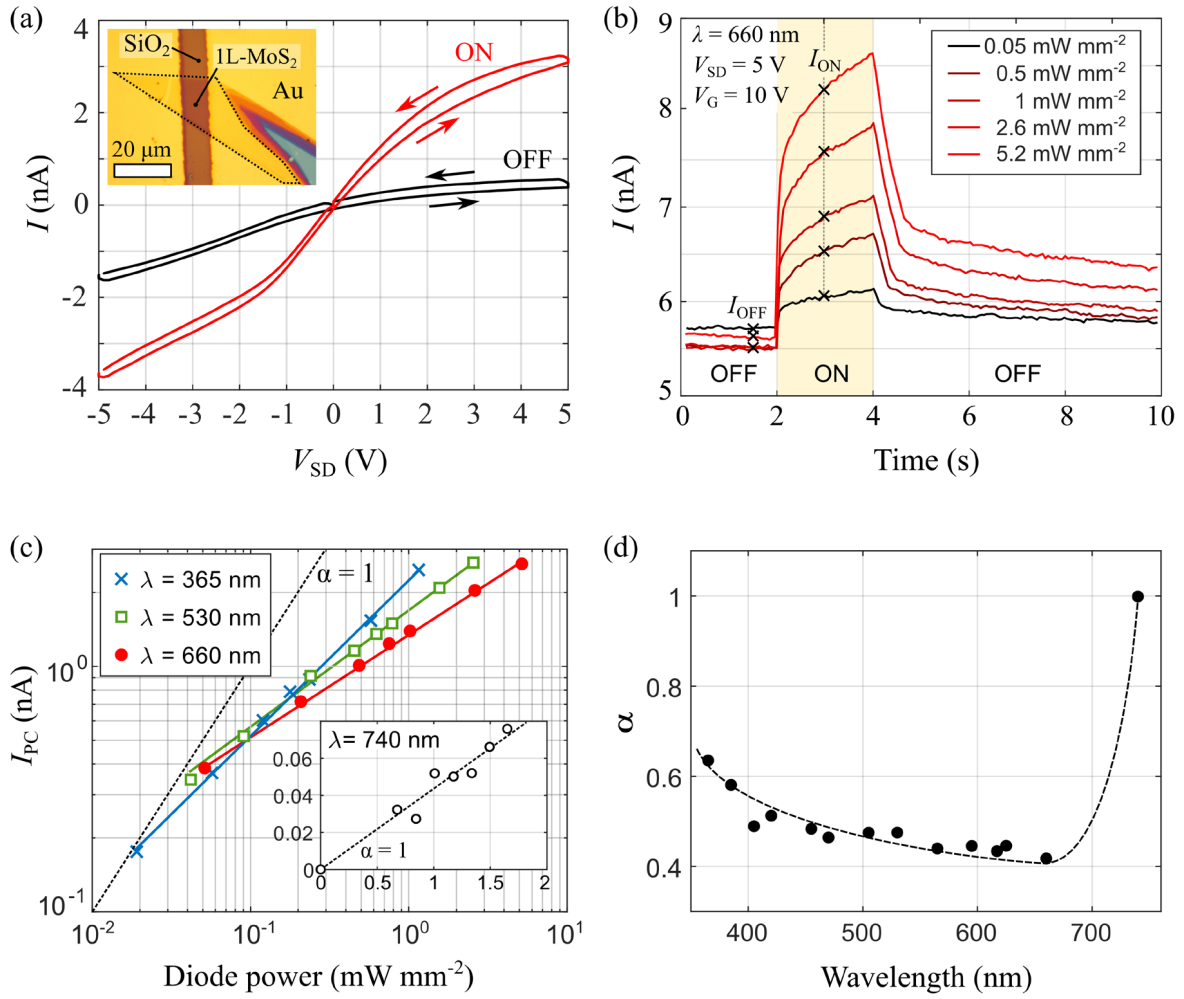
Optoelectronic response of the 1L-MoS _2_ transistor. ( **a**) *I-V* characteristics of the device at *V*_g_ = 0 V, measured in the dark (black) and upon illumination with *λ* = 660 nm and *P*_D_ = 5.2 mW mm ^–2^. Arrows indicate the voltage ramping direction. Inset: Optical image of the device. ( **b**) Drain-source current *I* measured at *V*_SD_ = 5 V and *V*_G_ = 10 V while switching the illumination on and off at different light power densities. ( **c**) Power dependence of the photocurrent *I*_PC_ at three different illumination wavelengths. *I*_PC_ is measured as the difference between the drain-source current one second after ( *I*_ON_) and immediately before ( *I*_OFF_) turning on the illumination. Solid lines are fittings to equation *I*_PC_ ∝ *P* ^α^. For reference, the slope corresponding to *α* = 1 is showed as a black, dashed line. ( **d**) Wavelength dependence of the *α* parameter, extracted from the fittings to Equation 1. The dashed line is a smoothed interpolation of the experimental data.

In the following, we demonstrate the capabilities of the LED light sources by performing a step-by-step characterization of a single-layer (1L) MoS _2_ phototransistor. The device is fabricated by standard mechanical exfoliation of monolayer MoS _2_ crystals with Nitto SPV-224PR-MJ tape and Gel-Film WF X4 (by Gelpak) tape, and ulterior transfer onto a SiO _2_/Si substrate with prepatterned Ti/Au electrodes, following the deterministic transfer technique described in references ^16– 18^. The resulting device is showed in the inset of Figure 2a ^19^. The main panel in Figure 2a shows two current *vs*. voltage ( *I-V* hereafter) characteristics of the 1L-MoS _2_ device, acquired in the dark (black) and under homogeneous illumination with *λ* = 660 nm, a power density *P*_D_ = 5.2 mW mm ^–2^ and a spot of 375 µm in diameter (red). The *I-V* curves are nonlinear due to the presence of Schottky barriers at the Au/MoS _2_ interfaces. Upon illumination, the drain-source current *I* increases by an amount *I*_PC_ due to photoconductivity.

Figure 2b shows the time evolution of the drain-source current, registered while turning the illumination on and off with a computer by using the external modulation port of the LEDD1 controller and the TENMA Programmable Bench Power Supply to turn on and off the LED source. As further discussed below, the optoelectronic response of the device is dominated by photogating, resulting in a slow photoresponse, which takes several seconds to stabilize after the light is switched on. The different curves shown in the figure correspond to consecutive measurements acquired for increasing illumination power densities. For completeness, we provide the Matlab script used to acquire these measurements on Zenodo ^20^. For each measurement, the sample is exposed to light for 2 seconds and then kept in the dark for 30 seconds to recover the original “off” current *I*_OFF_. The photocurrent *I*_PC_ is then calculated as the difference between the current *I*_ON_, registered 1 second after the light is turned on, and *I*_OFF_, measured immediately before exposure to light.

The measurements from Figure 2b allow us to estimate the response time of the device, as discussed in Supplementary Note 3 in the extended data ^21^. For our 1L-MoS _2_ device we get a rise time of *t*_R_ = 2.1 s and a much longer fall time *t*_F_ = 20.2 s.

Note that the possibility of adjusting the power density of the incident light with a software makes it possible to quickly measure the power dependence of the photogenerated current at many different wavelengths. This task can be very tedious for systems requiring the use of a manually-operated neutral density filter wheel to modify the incident power density.

In monolayer MoS _2_ phototransistors, photoconductivity typically originates from two main mechanisms: photogating and photoconductive effect. These two mechanisms can be distinguished by their different dependence on the illumination power density ^22– 24^. The typical procedure consists on fitting the measured photocurrent to the phenomenological equation

$$I_{PC}=R_{0}\times P^{\alpha },\;(1)$$

Where *R*_0_ and α are fitting parameters. As a general rule, α = 1 in devices which photoresponse is dominated by the photoconductive effect, and α < 1 for photogating ^22, 24– 27^. Figure 2c shows the power dependence of *I*_PC_ for different illumination wavelengths, *λ*. The resulting values of α are presented in Figure 2d as a function of *λ*. For illumination wavelengths lower than 700 nm (i.e. for photon energies larger than the optical bandgap of 1L-MoS _2_) *I*_PC_ depends sublinearly on the illumination power, and we get α ≈ 0.5. For *λ* > 700 nm the photoresponse decreases abruptly and the power dependence of *I*_PC_ becomes linear, suggesting a different photoresponse mechanism for sub-bandgap energy photons.

### Responsivity spectrum

**Figure 3.**
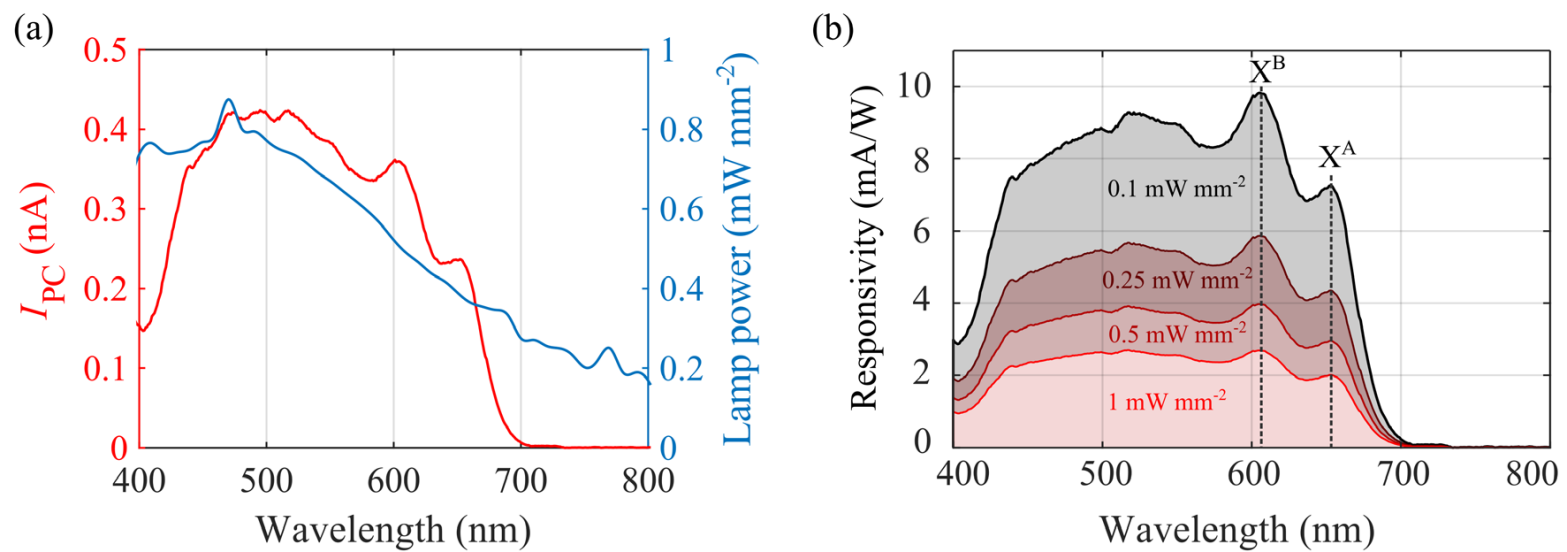
Responsivity spectra of the 1L-MoS _2_ device. ( **a**) Spectral dependence of *I*_PC_ (red line, left axis) and *P*_lamp_(blue line, right axis) ( **b**) Responsivity spectra calculated by inserting the experimentally measured values of *I*_PC_( *λ*), *P _lamp_* ( *λ*) and *α*( *λ*) into Equation 3 for four different values of the power density *P*.

The capability of the LED light sources to provide a spectrum of α as a function of wavelength ( Figure 2d) can also be exploited to correct the spectra measured with other light sources with non-flat spectral power density. Figure 3a shows a photocurrent spectrum of the 1L-MoS _2_ device, obtained using a wavelength-tunable Xenon light source (Bentham TLS120Xe). As also showed in the Figure, the spectral density of the light source, *P*_lamp_ ( *λ*), is not homogeneous throughout the whole spectral range, which introduces distortions in the measured spectral profile of *I*_PC_. For linear optoelectronic devices, where α = 1 (see Equation 1), one can trivially obtain the wavelength-dependent responsivity *R*( *λ*) from photocurrent spectra such as the one from Figure 3a by simply dividing *I*_PC_ by the spectral density of the lamp *P*_lamp_ ( *λ*) for each given wavelength. However, the situation is more complex for α ≠ 1, as in this case the responsivity depends nonlinearly on the illumination power:


$$R(\lambda ,P)\;=\;\frac{I_{PC}(\lambda )}{P}\;=\;R_{0}(\lambda )\;\times \;P^{\alpha (\lambda )-1}.\;(2)$$

Thus, after measuring *I*_PC_ as a function of *λ*, one also needs to know the wavelength dependence of the power α, which can be easily done using the presented LED light sources. Then, the device responsivity (at a given power *P*) can be obtained as 


$$R(\lambda ,P)\;=\;\frac{I_{PC}(\lambda )}{P}\;{\left(\frac{P}{P_{lamp}(\lambda )}\right)}^{\alpha (\lambda )}.\;(3)$$

Figure 3b shows the resulting responsivity spectra for four different illumination power densities *P* = 0.1, 0.25, 0.5 and 1 mW mm ^-2^. Note that, since in our device we get *α* < 1, the responsivity is larger for smaller values of *P*.

**Figure 4.**
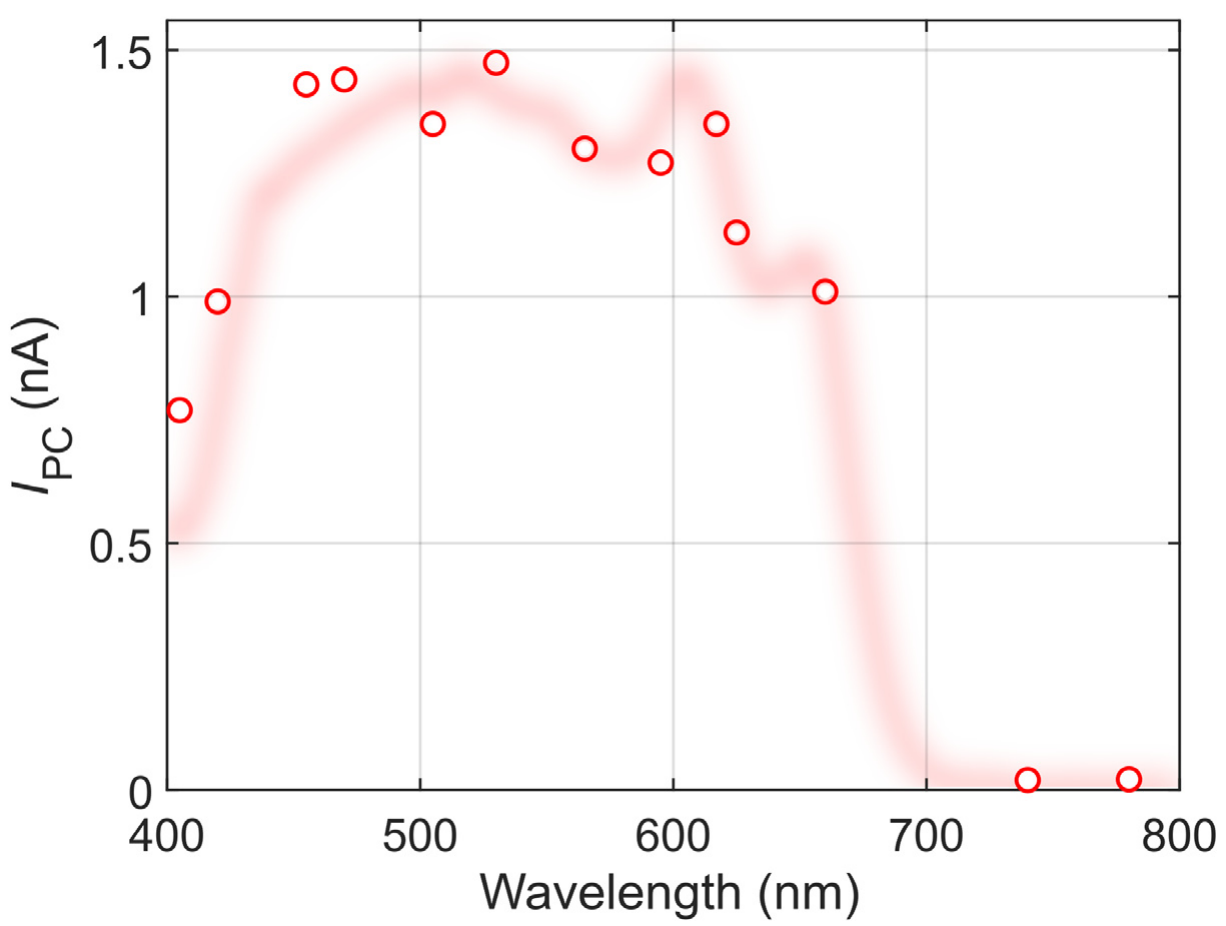
Photocurrent pseudospectrum of the 1L-MoS _2_ device, measured using the LED light sources at a fixed power density of 1 mW mm ^-2^, as a function of their nominal wavelength. The shaded red line shows the full photocurrent spectrum as a guide to the eye.

Note that the fiber-coupled LEDs can also be used as a standalone light source to characterize the basic spectral behavior of a given material/device. As an example, Figure 4 shows the wavelength dependence of the photocurrent in the 1L-MoS _2_ device, measured using the different LEDs at a fixed power density of 1 mW mm ^-1^. The resulting pseudospectrum could be used, for example, to estimate the absorption edge of 1L-MoS _2_ within ~ 30 nm accuracy.

### Characterization of device detectivity

**Figure 5.**
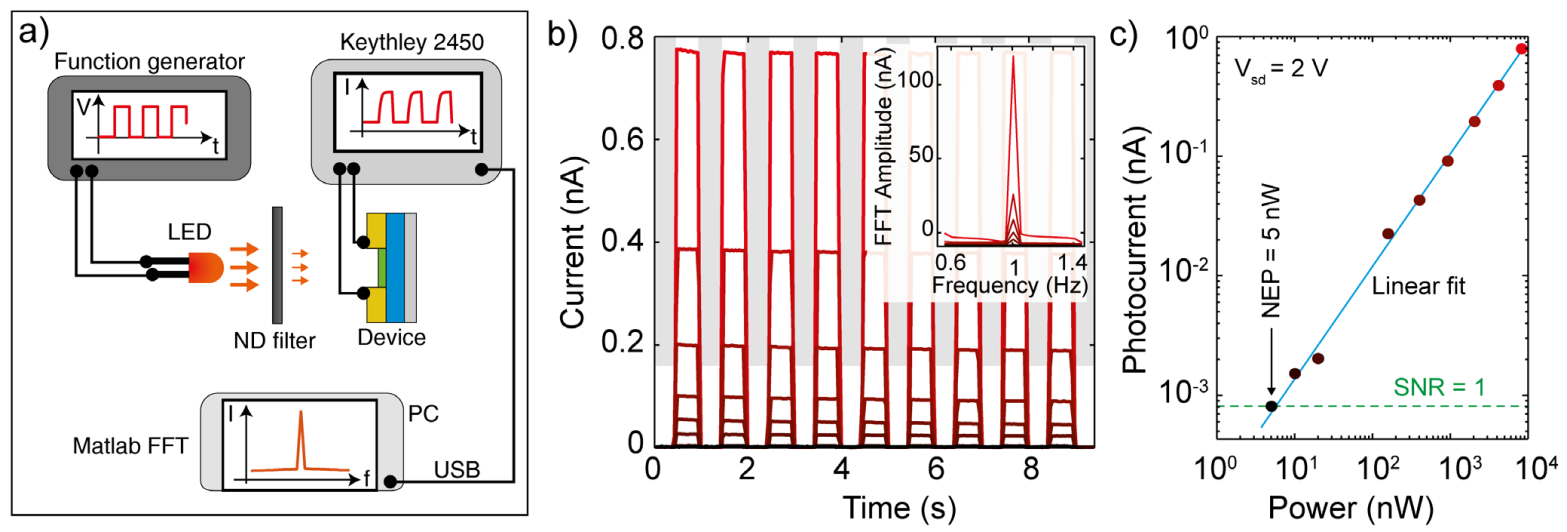
( **a**) Schematic of the experimental configuration to extract the detectivity of a device. ( **b**) Current versus time traces recorded on a Au-InSe-Au photodetector at a source drain voltage of 2 V while applying a 660 nm square wave modulated illumination with frequency 1 Hz. The different colors represent different illumination powers (achieved by reducing the intensity of the excitation using ND filters. Inset: fast fourier transform (FFT) of the current vs time traces. ( **c**) Photocurrent of the device (extracted from the amplitude of the FFT at 1 Hz) as a function of illumination power. The blue line is a linear fit to the data and the green dashed line indicates the noise level of the setup, which correspond to a signal-to-noise ratio (SNR) equal to 1.

Apart from the responsivity, another figure-of-merit widely used in the characterization of photodetectors is the specific detectivity D* ^26, 28, 29^. This quantity characterizes the performances of a photodetector in detecting small signals. Figure 5a shows a schematic of the experimental setup used to assess this figure-of-merit. We connect a function generator to a LED source and modulate the light intensity through a square wave, effectively turning on and off the illumination at a certain frequency ν (typically 1 Hz). We then shine the light onto the device under test and use a set of ND filters (from OD 0.3 to OD 4.0) to vary the intensity of the light. We record a set of current vs time traces, one for each illumination power. By computing the fast fourier transform (FFT) of each trace in Matlab, we can extract the response of the device at that power by extracting the amplitude of the FFT at the frequency ν. From this one can find the noise equivalent power of the device ( *NEP*), which can then used to calculate the specific detectivity $D^{*}=\frac{\sqrt{Af}}{NEP},$ where *A* is the area of the device and *f* is the bandwidth.

Figure 5b shows a set of current vs time traces recorded on a back-to-back Schottky diode realized by transferring an InSe multilayer flake bridging two gold electrodes deposited onto a SiO _2_ –Si substrate (see Supporting Note 4 for a picture of the device). The curves have been measured by applying a source drain voltage of 2 V while turning on and off the 660 nm illumination with a frequency of 1 Hz and different illumination powers (from 10 μW, red curve, to 5 nW, black curve). The fourier transforms of these traces, shown in the inset of Figure 5b, present a series of peaks at the odd harmonics of the fundamental frequency of 1 Hz. By extracting the area of the 1 Hz peak and plotting it as a function of the illumination power we can extract the NEP of our device. Figure 5c shows the photocurrent amplitude extracted from the FFT transforms in Figure 5b as a function of illumination power in a double logarithmic plot. The data follow a linear trend indicating that the photocurrent and the power are related by a power law dependency. The dashed line indicates the photocurrent intensity at which the signal to noise ratio (SNR) becomes equal to 1, which in our measurement corresponds to approximately 0.8 pA. The intersection between the linear fit and the line of unitary SNR happens at the power corresponding to the NEP of our device, which in this case is 5 nW. By applying the formula for the detectivity and using an area *A* = 1800 μm ^2^ and a bandwidth of 1 Hz, we find D* = 10 ^4^ Jones.

## Conclusions

In all, the LED-based light sources described here represent a low-cost, modular, and easy to use alternative to the commonly used free-space light sources. While the achievable spectral resolution of these light sources is limited, they still allow to extract the essential figures of merit of photodetector devices such as response time, responsivity, optical absorption edge and detectivity. The stability, homogeneous power density and absence of speckle makes these light sources especially well-suited for characterizing the power dependence of photoresponse in optoelectronic devices. Furthermore, they can even be used in combination with low-cost power sources for automated measurement. Thus, we believe that these light sources are an excellent alternative for performing optoelectronic characterization experiments on a limited budget.

## Data availability

### Underlying data

Zenodo: Fiber-coupled LEDs as safe and convenient light sources for the characterization of optoelectronic devices. https://doi.org/10.5281/zenodo.5153604 ^19^.

### Extended data

Zenodo: Supplementary Information to: Fiber-coupled LEDs as safe and convenient light sources for the characterization of optoelectronic devices. https://doi.org/10.5281/zenodo.5166973 ^21^.

This project contains the following extended data:

- AlphaSpectroscopy_SuppInfo_v7.pdf (Supplementary Notes 1-4)

Data are available under the terms of the Creative Commons Attribution 4.0 International license (CC-BY 4.0).

### Analysis code

Analysis code for the ON-OFF measurements presented in Figure 2b available at: https://github.com/JorgeQuereda/Fiber-coupled-LEDs-as-safe-and-convenient-light-sources-for-the-characterization-of-optoelectronic-d/tree/v1.0

Archived analysis code as at time of publication: https://doi.org/10.5281/zenodo.5153596 ^20^.

License: Creative Commons Zero "No rights reserved" data waiver (CC0 1.0 Public domain dedication)
