# Supplementary material for: Fiber-coupled light-emitting diodes (LEDs) as safe and convenient light sources for the characterization of optoelectronic devices
Source: Open Res Eur. 2022 Jul 13;1:98. Originally published 2021 Aug 23. [Version 2] doi: 10.12688/openreseurope.14018.2 (PMC10446081; doi:10.12688/openreseurope.14018.2)
Supplement: Peer review history

# Peer review: Reviewer response for version 2

**DOI:** 10.21956/openreseurope.16034.r29781

**Published:** 2022-07-29

**Authors:** Marcel Claro

**Affiliations:** 1 Universidade de Santiago de Compostela, Santiago de Compostela, Spain

The manuscript "Fiber-coupled light-emitting diodes (LEDs) as safe and convenient light sources for the characterization of optoelectronic devices " demonstrates the use of a combination of LEDs with different wavelengths for optoelectronic characterization of photodetectors and/or photoresponsive materials, in substitution of conventional white light+monochromator setup or lasers. It gives some application examples for 2D material photodetectors. It presents a very practical, robust and relatively cheap setup which I would recommend for this application. All my questions in the 1st version were answered and I recommend for indexing as it is.

Is the rationale for developing the new method (or application) clearly explained?

Yes

Is the description of the method technically sound?

Partly

Are the conclusions about the method and its performance adequately supported by the findings presented in the article?

Partly

If any results are presented, are all the source data underlying the results available to ensure full reproducibility?

Yes

Are sufficient details provided to allow replication of the method development and its use by others?

Yes

Reviewer Expertise:

Molecular beam epitaxy, III-V, II-VIs, and 2D materials, optoelectronic device fabrication and characterization, mid-infrared.

I confirm that I have read this submission and believe that I have an appropriate level of expertise to confirm that it is of an acceptable scientific standard.

# Peer review: Reviewer response for version 1

**DOI:** 10.21956/openreseurope.15106.r27993

**Published:** 2021-11-05

**Authors:** Marcel Claro

**Affiliations:** 1 Universidade de Santiago de Compostela, Santiago de Compostela, Spain

The manuscript "Fiber-coupled light-emitting diodes (LEDs) as safe and convenient light sources for the characterization of optoelectronic devices " demonstrates the use of a combination of LEDs with different wavelengths for optoelectronic characterization of photodetectors and/or photoresponsive materials, in substitution of conventional white light+monochromator setup or lasers. It gives some application examples for 2D material photodetectors. The paper is well written and presents valuable information.

 Here are my comments.

1. Considering the price for LED+controller and the number of LEDs required, it would not be much cheaper than lamp + monochromator kit. The practicality is an obvious aspect, but I would also expect some gain in the light intensity in specific wavelengths (where filtered white light is usually weak). Then it would be great to have some comments on:
  a) Table with specifications (resolution, intensity at some wavelengths, wavelength range) and direct comparison with setup with lamp+monochromator in a similar price range, compare price.
  b) The necessity of dedicated LED controllers for each LED, the possibility of using a switching matrix or other solution to multiplex the LEDs, and use one controller to further reduce the price.
2. There are several unclear points in the detectivity section. It does not show any particularity of the light source system and could be removed. Some of the unclear points are:
  a)The bandwidth of 1Hz. Is it related to the FFT? sample rate (and then integration time)?
  b)What is the contribution of amplifier noise? Noise floor level?
  c)It would be great to have a full FFT spectrum in the SI, then comment on 1/f noise, especially at 1Hz.
  d)10 ^4^ Jones is extremely low for photodetectors (especially for visible wavelengths). Is it right? any comment on why it is so low?

I recommend the paper for indexing after solving these issues.

Is the rationale for developing the new method (or application) clearly explained?

Yes

Is the description of the method technically sound?

Partly

Are the conclusions about the method and its performance adequately supported by the findings presented in the article?

Partly

If any results are presented, are all the source data underlying the results available to ensure full reproducibility?

Yes

Are sufficient details provided to allow replication of the method development and its use by others?

Yes

Reviewer Expertise:

Molecular beam epitaxy, III-V, II-VIs, and 2D materials, optoelectronic device fabrication and characterization, mid-infrared.

I confirm that I have read this submission and believe that I have an appropriate level of expertise to confirm that it is of an acceptable scientific standard, however I have significant reservations, as outlined above.

# Response

**Published:** 2022-05-19

**Authors:** Jorge Quereda

Marcel Claro: Referee #2 Referee #2 (Reviewer comments in italics):

*The manuscript "Fiber-coupled light-emitting diodes (LEDs) as safe and convenient light sources for the characterization of optoelectronic devices " demonstrates the use of a combination of LEDs with different wavelengths for optoelectronic characterization of photodetectors and/or photoresponsive materials, in substitution of conventional white light+monochromator setup or lasers. It gives some application examples for 2D material photodetectors. The paper is well written and presents valuable information. Here are my comments. Q1. Considering the price for LED+controller and the number of LEDs required, it would not be much cheaper than lamp + monochromator kit. The practicality is an obvious aspect, but I would also expect some gain in the light intensity in specific wavelengths (where filtered white light is usually weak). Then it would be great to have some comments on:*

*1.a) Table with specifications (resolution, intensity at some wavelengths, wavelength range) and direct comparison with setup with lamp+monochromator in a similar price range, compare price.*

**Response**: This is a good point. Regarding the price, as stated in your point 1b, the cost could be reduced drastically (see below). Regarding the point about the higher intensity of the LEDs with respect to lamp+monochromator systems, we have now compared the power intensity out of a 400 µm core multimode fiber (NA 0.39) when using the LEDs and when using a xenon lamp + monochromator system of the same price range (~15 k€) and similar linewidth output (Bentham TLS120Xe; average FWHM of ~12 nm). We have included this comparison in the Supporting Information.

*1.b) The necessity of dedicated LED controllers for each LED, the possibility of using a switching matrix or other solution to multiplex the LEDs, and use one controller to further reduce the price.*

**Response:** This is an excellent point that we didn’t discuss in the original manuscript. That is completely feasible, one could easily control all the LEDs using a unique controller and a switch box or even connecting the LEDs directly to the benchtop programmable power supply that we use to modulate the power (TENMA 72-2705). This would reduce the cost from ~800€ to ~450€ per wavelength. In the revised version of the text, we now comment on this possibility to make the system more affordable.

*Q2. There are several unclear points in the detectivity section. It does not show any particularity of the light source system and could be removed. Some of the unclear points.*

**Response:** We wanted to demonstrate that this simple illumination system could be used to directly determine experimentally the detectivity, as in the literature of 2D materials it is very common to use indirect methods to determine the detectivity, which in some cases could lead to a gross overestimation of the detectivity. We have seen in other groups that quite sophisticated setups with dedicated acquisition electronics are used to measure the detectivity and thus we believed that it might provide valuable information to show how this measurement can also be done at a lower price tag with the LED sources.

*2.a) The bandwidth of 1Hz. Is it related to the FFT? sample rate (and then integration time)?*

**Response**: The sampling rate of the measurement is 50 Hz (integration time of 20 ms), while the square wave modulated light illumination frequency is 1 Hz, according to the definition of the detectivity (see, for example, https://www.nature.com/articles/s41566-018-0288-z or https://www.thorlabs.com/images/TabImages/Noise_Equivalent_Power_White_Paper.pdf).

*2.b) What is the contribution of amplifier noise? Noise floor level?*

**Response: **The data acquisition electronics (including current to voltage amplifiers) can contribute to the measurement of detectivity through their noise floor level, i.e. the total noise of the system (device + amplifier) is determined by the sum of the squares of the various noise components. For our Keithley 2450 at a current range of 10 nA, the current noise level in the 1 Hz frequency region is smaller than 0.2 pA. Thus, the noise floor observed in the experiment is due mostly to the device noise. In the experiment, the illumination power at which one reaches the level of SNR = 1 gives the noise-equivalent-power of the system, allowing to determine the corresponding detectivity.

*2.c) It would be great to have a full FFT spectrum in the SI, then comment on 1/f noise, especially at 1Hz.*

**Response:** We now present the FFT of the dark trace (shutter closed and LED still modulated at 1 Hz) in the Supplementary Information. We find a low frequency region with power law exponent of -0.5 and a higher frequency component of -2.5 indicating that there are other noise generating mechanisms that go beyond the 1/f noise.

*2.d) 104 Jones is extremely low for photodetectors (especially for visible wavelengths). Is it right? any comment on why it is so low?*

**Response:** Using the commonly used formula of the detectivity based on the assumption of a shot noise limited photodetector we find a detectivity of 109 Jones. The value that we give in the main text is a lower boundary of the detectivity since we did not measured directly the bandwidth and used the lowest value of 1 Hz. Note that the value that we previously reported was also low because of an error in the calculation, the area value used was in meters squared instead of centimeters squared. The correct value is 107 Jones. We thank the reviewer for highlighting this error, which has been corrected in the revised manuscript.

# Peer review: Reviewer response for version 1

**DOI:** 10.21956/openreseurope.15106.r27466

**Published:** 2021-10-11

**Authors:** Antonio Rossi

**Affiliations:** 1 Department of Physics and Astronomy, University of California, Davis, Davis, CA, USA

The manuscript "Fiber-coupled light-emitting diodes (LEDs) as safe and convenient light sources for the characterization of optoelectronic devices " describe a cost-effective and portable setup for multi-wavelength device characterization. The setup is built using a modular selection of light-emitting diodes and it is presented as an alternative to conventional and more expensive light source. The authors report on the optoelectronic characterization of a photodetector made from transition metal dichalcogenide MoS2. The paper is well written and offers detailed guidelines for reproducing the results shown.

 Here below, my comments to the paper are reported.

1. In the introduction the authors state that they have been using such a setup for 7 years. Is there any peer reviewed work that has been published with the support of such a setup? If yes, I would suggest to report on that in the introduction.
2. At the end of the introduction the authors claim that the presented fiber-coupled LEDs are rather inexpensive if compared to more sophisticated laser systems, underlining the modular capability of the setup. Is this the only advantage with respect a traditional light source? What is the downside of not using a coherent laser? What are the types of characterization you cannot perform? For users approaching this technique for the first time, it would be useful to understand what they can but also what they cannot do.
3. In the methods section the authors write that the spectral line F-W-H-M of the single light source can range from 10 to 100 nm. Figure 1B also supports this claim, where the 595 and 565 nm lights seem to be very broad, together also with the 385 nm one. In a number of measurements the authors show wavelength dependent results. In particular when discussing photocurrent, responsivity but also detectivity of the device. How reliable is a source with a FWHM of 100 nm when extracting this figures of merit?
4. In Figure 2(b) the authors show the drain source current I measured on a ON-OFF cycle. The authors identify the photogating effect as the main cause of the long rump-up till saturation and the slow recovery of the dark state. Would it be possible to provide at least one single curve that shows the system completely recovering the OFF state? Can the authors comment on what is causing the photogating?

In conclusion, I recommend the paper for indexing after addressing my comments above.

Is the rationale for developing the new method (or application) clearly explained?

Yes

Is the description of the method technically sound?

Partly

Are the conclusions about the method and its performance adequately supported by the findings presented in the article?

Partly

If any results are presented, are all the source data underlying the results available to ensure full reproducibility?

Yes

Are sufficient details provided to allow replication of the method development and its use by others?

Yes

Reviewer Expertise:

My area of expertise is on 2D materials, x-ray photoemission and optical spectroscopy, nano-fabrication and scanning probe microscopy.

I confirm that I have read this submission and believe that I have an appropriate level of expertise to confirm that it is of an acceptable scientific standard.

# Response

**Published:** 2022-05-19

**Authors:** Jorge Quereda

We would like to thank the Referees Antonio Rossi and Marcel Claro for providing constructive comments and suggestions and for their positive appraisal of our work. We have accordingly modified the manuscript adding the changes noted.

 Antonio Rossi: Referee #1 Referee #1 (Reviewer comments in italics): *The manuscript "Fiber-coupled light-emitting diodes (LEDs) as safe and convenient light sources for the characterization of optoelectronic devices " describe a cost-effective and portable setup for multi-wavelength device characterization. The setup is built using a modular selection of light-emitting diodes and it is presented as an alternative to conventional and more expensive light source. The authors report on the optoelectronic characterization of a photodetector made from transition metal dichalcogenide MoS2. The paper is well written and offers detailed guidelines for reproducing the results shown.*

*Here below, my comments to the paper are reported Q1. In the introduction the authors state that they have been using such a setup for 7 years. Is there any peer reviewed work that has been published with the support of such a setup? If yes, I would suggest to report on that in the introduction.*

**Response:** Thank you for raising this point. As a matter of fact, we have used different versions of this setup along the last years in some of our peer-reviewed works. We now include references to those works.

*Q2. At the end of the introduction the authors claim that the presented fiber-coupled LEDs are rather inexpensive if compared to more sophisticated laser systems, underlining the modular capability of the setup. 2.a) Is this the only advantage with respect a traditional light source?*

**Response:** Apart from the low cost and the modular design, we believe that fiber-coupled LEDs have other advantages. In particular, as now stressed in the revised manuscript, their improved safety in comparison with free-space laser systems, as well as their capability to modulate the light power and/or switch it ON/OFF with an external voltage, without need of neutral density filters nor mechanical choppers.

*2.b) What is the downside of not using a coherent laser?*

**Response: **Free-space laser sources typically have higher intensity and narrower linewidths.

*2.c) What are the types of characterization you cannot perform?*

**Response:** These light sources are not adequate for fine spectroscopic measurements requiring an exquisite energy/wavelength resolution (e.g. photocurrent spectroscopy of samples with narrow features like excitonic peaks).

*2.d) For users approaching this technique for the first time, it would be useful to understand what they can but also what they cannot do.*

**Response:** We agree with this point and in the revised version of the manuscript we now discuss the limitations of this setup in line with what has been discussed above: “The presented fiber-coupled LEDs are rather inexpensive, if compared with laser systems, and are modular, making it possible to improve the system little by little. While these light sources cannot compete with lasers in terms of spectral narrowness and output power, their improved safety, as well as their capability to modulate the light power and/or switch it ON/OFF with an external voltage, without the need of neutral density filters nor mechanical choppers, make them very flexible light sources, suitable for characterization techniques where a large output power and extremely narrow bandwidth are not a must.”

*Q3. In the methods section the authors write that the spectral line F-W-H-M of the single light source can range from 10 to 100 nm. Figure 1B also supports this claim, where the 595 and 565 nm lights seem to be very broad, together also with the 385 nm one. In a number of measurements the authors show wavelength dependent results. In particular when discussing photocurrent, responsivity but also detectivity of the device. How reliable is a source with a FWHM of 100 nm when extracting this figures of merit?*

**Response:** It is true that some of the LEDs have a much wider spectral line and it means that the spectral features and the figures of merit extracted with those LEDs will be averaged over a wider wavelength/energy range. This will be a limitation when probing a system with sharp spectral features around those wavelengths. In that case one could mitigate this limitation by coupling narrow band-pass filters at the output of the optical fiber to reduce the FWHM to 10 nm. In the revised version of the manuscript we now comment on this limitation and the possible way to tackle it.

*Q4. In Figure 2(b) the authors show the drain source current I measured on a ON-OFF cycle. The authors identify the photogating effect as the main cause of the long rump-up till saturation and the slow recovery of the dark state. Would it be possible to provide at least one single curve that shows the system completely recovering the OFF state? Can the authors comment on what is causing the photogating?*

**Response: **We now include a dataset in the Supporting Information that shows a full recovery to the OFF state that takes more than 30 s.

*Q5. In conclusion, I recommend the paper for indexing after addressing my comments above.*

**Response:** Thank you for the appreciation, we tried to address the points raised as thoroughly as possible.
